# KLF9 in cancer: a potential prognostic marker and therapeutic target

**DOI:** 10.3389/fonc.2025.1630547

**Published:** 2025-08-11

**Authors:** Xiaoxiao Xiao, Zhipeng Dai, Fang Liu, Xingqi Zhao, Qiang Wu

**Affiliations:** ^1^ Faculty of Chinese Medicine, Hunan Traditional Chinese Medical College, Zhuzhou, China; ^2^ The State Key Laboratory of Quality Research in Chinese Medicine, Macau University of Science and Technology, Macao, Macao SAR, China; ^3^ Faculty of Rehabilitation and Health Care, Hunan Traditional Chinese Medical College, Zhuzhou, China; ^4^ School of Pharmacy, Binzhou Medical University, Yantai, China

**Keywords:** KLF9, cancer, transcription factor, signaling pathway, tumor microenvironment, epigenetic modification, therapeutic target

## Abstract

Kruppel-like factor 9 (KLF9) is a transcription factor that has gained significant attention in recent years for its critical involvement in development and progression of various cancers. Recent research has revealed the dual nature in tumorigenesis, where KLF9 can function as either a tumor suppressor or an oncogene, depending on the cellular context. Clinically, KLF9 emerges as a potential prognostic biomarker due to its differential expression patterns across various cancer types, with lower KLF9 levels often correlating with poorer patient outcomes. Furthermore, KLF9 represents a promising therapeutic target, as modulating its activity may offer new strategies for cancer treatment. Overall, the present review provides new insights and perspectives for future research on KLF9 in cancer, underscoring its importance in personalized medicine.

## Introduction

The Krüppel-like factor (KLF) family of transcription factors, characterized by conserved zinc finger DNA-binding domains, play pivotal roles in diverse biological processes ranging from cellular differentiation to tumorigenesis (1). KLF members are now understood to exhibit distinct expression patterns and functional roles in tumorigenesis across various cancers. For instance, KLF4 is often associated with tumor suppression (2), while KLF5 has been linked to promoting tumor growth in breast cancer (3). Besides, KLF9 (also known as Basic Transcription Element-Binding Protein 1, BTEB1) has emerged as a critical regulator in both physiological and pathological contexts. Initially identified as a transcriptional repressor of cytochrome P450 genes, KLF9 has since been implicated in cell cycle regulation, apoptosis, and tissue development (4). However, its dysregulation in cancer has attracted significant interest over the past decade, with accumulating evidence highlighting its context-dependent roles as either a tumor suppressor (5).

### Biological Features of KLF9

KLF9, located on human chromosome 9q13, encodes a 244-amino acid protein containing three C-terminal C2H2 zinc fingers that bind to GC-rich promoter regions (6). Structurally, KLF9 shares homology with other KLF family members, particularly KLF13, KLF14 and KLF16, but exhibits unique regulatory properties due to its N-terminal repression domain (1, 7). KLF9 modulates gene expression through multiple mechanisms, including recruitment of co-repressors such as Sin3A/HDAC complexes and competition with activating transcription factors for promoter occupancy (8). Physiologically, KLF9 modulates critical processes such as endometrial cycling, neuronal differentiation, and metabolic homeostasis. For instance, KLF9-knockout mice displayed impaired uterine decidualization and fertility defects, underscoring its role in reproductive biology (9). In the brain, KLF9 regulates dendritic arborization and synaptic plasticity, with its deficiency linked to neurodevelopmental disorders (10). These diverse roles stem from KLF9’s ability to integrate hormonal signals (e.g., progesterone, thyroid hormone) and environmental cues into transcriptional programs that maintain cellular quiescence or drive differentiation (11).

KLF9’s expression patterns show significant correlation with patient prognosis across various cancer types, with low expression often indicating poor outcomes, highlighting its potential as a prognostic biomarker. Furthermore, modulating KLF9 activity may offer novel therapeutic strategies, positioning it as a promising treatment target. This comprehensive review aims to consolidate recent advances in KLF9 research, which could facilitate the development of personalized medicine approaches for cancer patients. By synthesizing current knowledge, we seek to provide new perspectives and directions for future investigations in this evolving field.

## KLF9 in specific cancer types

Over the years, KLF9 has been implicated in various tumors, exhibiting heterogeneous expression levels and functional roles. In this section, a comprehensively review of the functional significance of KLF9 across major cancer types is conducted.

### Breast cancer

In breast cancer, KLF9 functions as a tumor suppressor, exhibiting inverse correlation with its expression and tumor aggressiveness. Studies have demonstrated significantly lower KLF9 expression in breast cancer tissues compared to benign tissues, which is associated with enhanced cell migration and invasion (12, 13). Mechanistically, KLF9 regulates the transcription of E-cadherin, a crucial protein for maintaining cell-cell adhesion, thereby inhibiting metastatic potential. Restoration of KLF9 expression in breast cancer cell lines has been shown to suppress their invasive capabilities, suggesting its therapeutic potential for preventing metastasis (14). Furthermore, the interactions between KLF9 with various microRNAs (such as miR-889) highlight its regulatory complexity and potential as a biomarker for breast cancer prognosis (13). Another study showed that KLF9 exerts bidirectionally regulation of core circadian clock genes, suggesting its role as a hormonal-circadian interface. Besides, KLF9 exhibits pan-subtype tumor-suppressive activity, while demonstrating context-dependent therapeutic modulation. Specifically, it potentiates GR-mediated anti-tumor effects in ER+ MCF7 luminal cells, while counteracting GR-driven oncogenicity in triple-negative breast cancer (TNBC) models (15). The difference in the role of KLF9 in ER+and TNBC may be due to the remodeling of its molecular behavior by subtype specific signal network and microenvironment background. In ER+breast cancer, KLF9 may enhance the anti-tumor effect by cooperating with GR, activate GR dependent growth suppressor genes (such as GILZ) and integrate estrogen signal feedback regulation to achieve proliferation inhibition; In TNBC, KLF9 directly inhibits the NF - κ B-MMP9 signaling axis through epigenetic silencing mechanisms (such as recruiting HDAC1 to the target gene promoter region) and antagonizes the GR driven pro cancer MAPK/STAT3 pathway, thereby inhibiting metastasis. The multidimensional regulatory basis of this functional polarization may include the open hormone responsive chromatin region in ER+cells, which makes KLF9 more prone to recruit co activated complexes, while the high methylation tendency of TNBC leads to transcriptional repression. And the tumor microenvironment (TME) background may further reinforce this difference. The adipocyte paracrine secretion of ER+tumors provides estrogen precursors to support the KLF9-GR anti-tumor axis, while the high inflammatory cytokine environment of TNBC activates NF - κ B to form antagonistic interactions with KLF9. KLF9 also plays an important role in the regulation of key signaling pathways, including those related to hormone signaling and cell cycle control, underscoring its importance in breast cancer biology. For example, KLF9 has been shown to enhance the expression of PTEN, a well-characterized tumor suppressor, thereby inhibiting aerobic glycolysis and reducing chemotherapy resistance in breast cancer cells (16). Bai et al. revealed that KLF9 inhibits breast cancer metastasis by transcriptionally repressing MMP9 and other NF-κB target genes through direct binding to the CACCC motif and subsequent recruitment of HDAC1 to silence their transcription (5). Clinical research has also demonstrated that reduced KLF9 expression in breast cancer tissues is correlated with poorer prognosis. Analysis of 68 paired tumor/normal samples revealed significantly lower KLF9 levels in cancerous tissues. Besides, patients with high KLF9 expression showed superior survival rates versus low expression counterparts (17). These studies overlap in their assertion of KLF9 as both a prognostic biomarker and potential tumor suppressor in breast cancer progression.

### Colorectal cancer

In colorectal cancer (CRC), KLF9 exhibits a tumor-suppressive role, evidenced by its downregulated expression in cancerous tissues compared to normal tissues (18). This reduced expression is associated with enhanced migratory and invasive capabilities of CRC cells, suggesting that KLF9 acts as a barrier against tumor progression. For example, the miR-135 family has been implicated in the regulation of KLF9, where increased levels of these microRNAs lead to decreased KLF9 expression, further promoting CRC cell proliferation and invasion (19). Another study revealed that the calcium-dependent enzyme PAD4 was upregulated in CRC through direct transcriptional activation by KLF9, promoting tumor growth and metastasis (20). KLF9 was identified as a key component of a 4-gene prognostic signature (MCM2, INHBA, CGREF1, KLF9) for early-onset colorectal cancer (EOCRC). The expression patterns and age-related prognostic value of KLF9 demonstrate its critical role in predicting survival outcomes. As an integral component of a robust predictive model (AUC 0.826-0.893 for 1–5 year survival), KLF9 further contributes to risk stratification by demonstrating significant correlation with DNA repair defects, PIK3CA mutations, and immune microenvironment alterations in EOCRC patients (21).

### Endometrial cancer

The role of KLF9 in endometrial cancer (EC) has attracted significant interest due to its association with tumor aggressiveness. Research indicates that KLF9 expression is significantly downregulated in EC tissues compared to normal endometrial tissues, and correlated with increased metastatic potential (22, 23). Besides, KLF9 can directly bind to the FBXO31 promoter to boost its transcription. Restoring KLF9 expression inhibited EC cell proliferation, invasion, and migration while increasing apoptosis and cisplatin sensitivity through FBXO31 upregulation. These findings collectively suggest that the KLF9-FBXO31 axis suppresses EC progression and enhances chemotherapy response, highlighting its dual therapeutic potential as both a tumor suppressor and chemosensitizer in EC management (24). Besides, it has been shown that KLF9 suppresses EC cell proliferation and invasion by antagonizing the Wnt/β-catenin signaling pathway (22). The progesterone receptor (PR) was found to transcriptionally activate KLF9 via direct promoter binding, with their expression levels positively correlated. These findings indicate that disruption of the PR-KLF9 regulatory axis contributes to EC progression (22). Frank A Simmen et al. revealed KLF9’s dual transcriptional roles in endometrial biology and cancer through genome-wide profiling in HEC-1-A cell sublines. Taken together, these findings establish KLF9 as a master regulator of uterine epithelial genes mediating coordinated activation/repression mechanisms that modulate pathways critical to both endometrial function (cell adhesion, differentiation) and carcinogenesis (motility, microenvironment remodeling) (25).

### Gastric cancer

In gastric cancer, KLF9 has been identified as a transcription factor that suppresses cell invasion and metastasis. Research has demonstrated that KLF9 expression is significantly reduced in metastatic gastric cancer tissues compared to non-metastatic counterparts. Mechanistically, KLF9 inhibits the transcription of matrix metalloproteinase 28 (MMP28), a protein that facilitates cancer cell invasion (26). KLF9 expression is also inhibited by direct targeting of miR-548d-3p at its 3’UTR, while KLF9 transcriptionally activates PER1 via promoter binding. Functionally, the TPTEP1/miR-548d-3p/KLF9/PER1 axis reduces gastric cancer cell migration and invasion, with KLF9 serving as the critical regulatory node that translates the long non-coding RNA TPTEP1’s tumor-suppressive effects into PER1-mediated anti-metastatic activity. This mechanistic cascade positions KLF9 as both a miRNA-regulated target and a transcriptional activator in gastric cancer progression (27). Furthermore, KLF9 contributes to FNDC5-mediated tumor suppression in gastric cancer. Current evidence suggests that FNDC5 expression positively correlates with KLF9, suggesting that KLF9 is involved in the epigenetic regulation (DNA methylation) of FNDC5. While not explicitly included in the prognostic risk score model, KLF9’s regulatory relationship with FNDC5 suggests its indirect contribution to the anti-metastatic effects and improved patient survival outcomes observed in the FNDC5-related prognostic framework (28).

### Hepatocellular carcinoma

In hepatocellular carcinoma (HCC), KLF9 reportedly plays a protective role against tumor progression. In KLF9-knockout mice fed a high-fat diet, hepatomegaly was observed independent of changes in adiposity parameters, accompanied by a significantly increase in hepatic oxidative stress markers and systemic oxidative imbalance. KLF9 deficiency could also amplify pro-inflammatory gene expression in the liver. These findings collectively reveal that KLF9 suppresses HCC risk factors by maintaining redox homeostasis and inhibiting inflammatory pathways, suggesting a broader tumor-suppressive function in cancers linked to metabolic dysfunction (29). Moreover, KLF9 expression is markedly reduced in metastatic HCC cells and clinical samples with metastases. KLF9 overexpression inhibited HCC migration and metastasis *in vitro*and *in vivo*, while its knockdown promoted these processes. Mechanistically, KLF9 directly binds to promoters of mesenchymal genes and repress epithelial-mesenchymal transition (EMT), while being reciprocally suppressed by the mesenchymal transcription factor Slug, forming a negative feedback loop. Clinically, KLF9 downregulation correlates with metastatic progression, establishing it as a therapeutically relevant transcriptional brake on HCC metastasis through bidirectional EMT control (30). Protein-protein interaction analysis revealed SAE1 could suppress KLF9 alongside other tumor suppressors while activating oncogenic pathways. Although mechanistic details of KLF9’s role remain unexplored in this context, its inclusion in SAE1-regulated tumor suppression networks suggests potential involvement in SAE1-driven HCC progression and metastasis (31). A study revealed that KLF9 could be suppressed via the AKT-EZH2 feedback loop in HCC progression (32). Collectively, KLF9 has emerged as a multifaceted tumor suppressor in HCC, counteracting oxidative stress, inflammation, EMT-driven metastasis, and epigenetic dysregulation. Its suppression via pathways like SAE1 and the AKT-EZH2-IGFBP4 axis underscores its central role in HCC pathogenesis. Restoring KLF9 function or targeting its regulatory networks (e.g., EZH2 inhibition) may offer novel therapeutic avenues to disrupt HCC progression and metastasis, warranting further exploration of its clinical potential.

### Non-small cell lung cancer

KLF9’s involvement in lung cancer, particularly non-small cell lung cancer (NSCLC), has been extensively studied, revealing its role as a tumor suppressor. KLF9 functions via directly regulating various microRNAs that influence tumor behavior, including miR-141, miR-660-5p, miR-625-3p and miR-889, which promote malignancy by targeting KLF9, suggesting that KLF9 could serve as a potential therapeutic target in NSCLC management (33–36). Besides, elevated KLF9 expression can reduce lung cancer cell proliferation, migration, and invasion by negatively regulating GADD34. *In vivo*, KLF9 silencing promotes tumor growth via GADD34-mediated recruitment of myeloid-derived suppressor cells (MDSCs), linking KLF9 loss to immunosuppression. These findings establish KLF9 as a crucial downstream effector of Mxi1, which functions as a negative regulator of Myc - a pivotal driver of tumorigenesis (37). KLF9 mediates tumor-suppressive effects by concurrently restraining intrinsic tumor malignancy and inhibiting the remodeling of immunosuppressive microenvironment components (38). Overall, KLF9 has emerged as a critical tumor suppressor in NSCLC, with its dysregulation contributing significantly to tumor progression and immune evasion. Hence, targeting the KLF9-microRNA axis or enhancing KLF9 activity might be a promising therapeutic strategy and warrants further investigation into its role in immunotherapy responses.

### Glioblastoma

KLF9 plays a multifaceted role in glioblastoma, particularly in modulating stemness and tumor aggressiveness. KLF9, when synergized with the histone deacetylase inhibitor panobinostat (LBH589), induces cell death in glioblastoma stem-like cells (GSCs). This combination can trigger apoptosis and necroptosis, reduce S-phase cell populations, and upregulate cell cycle inhibitors p21 and p27 (39). KLF9 also inhibited GSC tumorigenicity and stemness by suppressing integrin signaling via ITGA6 (40). In glioblastoma-derived neurospheres enriched with cancer stem cells, KLF9 was found to induce cell differentiation, suppress neurosphere formation, and inhibit xenograft growth by binding to the Notch1 promoter to repress Notch1 expression and downstream signaling. Notably, Notch1 pathway activation in GBM stem cells upregulates KLF9 expression, which correlates with reduced stemness markers (e.g., OLIG2) and growth rates. These findings highlight KLF9’s dual role as both a differentiation driver and a suppressor of Notch1-mediated oncogenic signaling in GBM stem cells, linking its activity to diminished tumor propagation and stem cell plasticity (41, 42). The dual therapeutic and mechanistic roles of KLF9 in glioblastoma underscore its potential role as a molecular switch to reprogram tumor stemness and therapy resistance. Future studies should explore strategies to pharmacologically enhance KLF9 activity or mimic its function, particularly in combination with HDAC inhibitors or Notch/ITGA6-targeted therapies.

### Ovarian cancer

In ovarian cancer (OC), KLF9 has been identified as a critical transcription factor that regulates tumor progression. Current evidence suggests that KLF9 can inhibit the proliferation and metastasis of OC cells by regulating various signaling pathways and microRNAs, including miR-600, which directly targets KLF9 (43). In OC patients, KLF9 expression was significantly reduced in blood samples compared to controls, suggesting its potential role as a tumor suppressor. KLF9 downregulation, alongside elevated TPD52 and miR-223 levels, correlated with disease progression and metastasis, positioning KLF9 as a promising blood-based prognostic biomarker and therapeutic monitoring tool in OC (44). In advanced OC ascites-derived tumor spheroids, KLF9 was downregulated and inversely correlated with cancer stemness, metastasis, and poor prognosis. Mechanistically, KLF9 suppressed stem-like properties by binding to the Notch1 promoter, inhibiting Notch1/Slug signaling. Interestingly, KLF9 was upregulated in primary OC tissues, and its lentivirus-mediated knockdown inhibited cell proliferation, induced G0/G1 arrest, and reduced tumor growth in xenografts. The opposite effect of KLF9 in primary and metastatic ovarian cancer may be attributed to molecular reprogramming driven by TME: in primary tumors, KLF9 may be shaped by the local microenvironment (such as matrix interactions or mechanical stress) as a pro survival factor, supporting cell proliferation through an unclear gene regulatory network, thus its knockout significantly inhibits tumor growth; In metastatic microenvironments such as ascites tumor balls, hypoxia, inflammatory signals, and three-dimensional structural pressure trigger the anticancer function of KLF9, which inhibits tumor stemness and invasiveness by directly suppressing the Notch1/Slug signaling axis. The underlying mechanism of this functional reversal may involve multidimensional regulation, such as differential methylation or histone modification of the KLF9 target gene promoter in primary and metastatic lesions; Differences in paracrine signaling pathways and spatiotemporal specific switching of KLF9 protein interaction network in tumor associated cells. The core contradiction lies in the binary plasticity of KLF9 as an “environmental sensor”. The microenvironment reshapes its molecular niche and pushes it towards opposite functional poles, serving as both a survival support factor for primary tumors and a stemness inhibitor in metastatic lesions.These findings collectively suggest that KLF9 acts as a tumor suppressor in metastatic niches by countering stemness but may paradoxically support tumor cell survival in primary sites, highlighting its potential as a therapeutic target and prognostic marker in OC (45, 46). While KLF9 acts as a tumor suppressor in metastatic niches by suppressing cancer stemness and Notch1/Slug-driven aggressiveness, its paradoxical upregulation in primary tumors suggests a microenvironment-specific duality. Future studies should elucidate the molecular mechanisms underlying KLF9’s dual behavior and validate its clinical utility for stratified treatment strategies in OC.

### Multiple myeloma

In multiple myeloma, KLF9 has been implicated in regulating tumor cell behavior through its interactions with long non-coding RNAs and microRNAs. The lncRNA DANCR has been identified as a negative regulator of KLF9, suggesting a complex regulatory network where KLF9 functions as a tumor suppressor (47). Multiple myeloma patients responding to the proteasome inhibitor bortezomib exhibited higher basal KLF9 expression. Both bortezomib and the HDAC inhibitor LBH589 (panobinostat) could upregulate KLF9 via HDAC inhibition. Mechanistically, KLF9 could bind to the promoter of the proapoptotic gene NOXA and drive its expression. Knockdown of KLF9 impaired bortezomib- or LBH589-induced NOXA upregulation and apoptosis, while KLF9 overexpression triggered partial NOXA-dependent apoptosis. These findings position KLF9 as a clinically relevant mediator of therapeutic responses in multiple myeloma (48). A genomic region of homozygosity (ROH) at chromosome 9q21, containing KLF9, showed a consistent association with multiple myeloma risk in genome-wide analyses. This KLF9-associated ROH emerged as a potential candidate risk locus warranting further investigation (49). Collectively, KLF9 plays its role in multiple myeloma as both a mediator of therapeutic response (via HDAC inhibitor-driven NOXA activation) and a potential genetic risk marker linked to the 9q21 ROH locus. Further studies should clarify the functional impact of KLF9-associated genomic homozygosity on transcriptional regulation and drug sensitivity profiles, while exploring whether KLF9 itself or its regulatory partners could serve as therapeutic targets to enhance proteasome inhibitor efficacy or overcome resistance mechanisms.

### Renal cell carcinoma

In renal cell carcinoma (RCC), KLF9 serves as a critical regulator of cell proliferation, migration, and invasion (50). The miR-140-5p/KLF9 axis has been identified as a key regulatory pathway, where miR-140-5p promotes RCC progression by targeting KLF9. Accordingly, restoring KLF9 expression or inhibiting miR-140-5p may offer therapeutic benefits in treating RCC (51). Current evidence suggests that KLF9 can directly bind to the SNX5 promoter and enhance its transcription in clear cell RCC. The KLF9-SNX5 axis suppresses TGF-β-induced EMT and metastasis, and combined analysis of KLF9 with SNX5, CD44, or E-cadherin improves prognostic accuracy in clear cell RCC. These findings highlight KLF9 as a critical regulator of SNX5-mediated tumor suppression, positioning the KLF9-SNX5 pathway as a potential therapeutic target in clear cell RCC (52). In kidney renal clear cell carcinoma (KIRC), KLF9 was found to be downregulated and associated with shorter overall survival. KLF9 was also found to correlate with immune cell infiltration and interact with immune-related genes, suggesting its role in modulating the tumor microenvironment (53).

The complex role of KLF9 in different cancer types has been summarized in **Table 1**. Notably, KLF9 contributes to cancer development in other malignancies, such as pancreatic cancer (54–56), prostate cancer (57), thyroid cancer (58–60), and bladder cancer (61–63). These studies collectively demonstrate that KLF9 plays multifaceted role as a tumor suppressor, epigenetic modulator, and therapeutic response mediator across diverse malignancies. While KLF9 consistently inhibits oncogenic processes, such as EMT, cancer stemness, and immune evasion, its regulatory networks exhibit tissue-specific complexity, governed by microRNAs (miRNAs), long non-coding RNAs (lncRNAs), and post-translational modifications. The dual context-dependent roles of KLF9 in cancers like ovarian and triple-negative breast cancer highlight the need for precision approaches when targeting KLF9 or its interactors. Future research should prioritize elucidating the molecular mechanisms underlying KLF9’s functional duality, optimizing strategies to restore its expression, and exploring combinatorial therapies that leverage KLF9’s synergy with epigenetic drugs or immune modulators. Clinically, validating KLF9 as a prognostic biomarker or therapeutic target requires robust multi-omics integration, particularly to decipher its crosstalk with tumor microenvironments and metabolic pathways. Bridging these mechanistic insights with translational efforts could unlock KLF9’s full potential as a linchpin in next-generation cancer therapeutics.

**Table 1:** Expression profiles, molecular mechanisms, and clinical implications of KLF9 in various cancers.

Cancer type	Expression	Function	Key mechanisms	Related molecules/pathways	Clinical relevance	References
Breast Cancer	Downregulated	Tumor suppressor	Regulates E-cadherin to inhibit metastasis	miR-889, GR, PTEN, MMP9	High KLF9 correlates with better survival (P<0.001)	(12–17)
			Enhances PTEN expression			
Colorectal Cancer	Downregulated	Tumor suppressor; prognostic marker	Part of 4-gene prognostic model (MCM2/INHBA/CGREF1/KLF9)	miR-135, PAD4, DNA repair pathways	Predicts survival in early-onset CRC (AUC 0.826–0.893)	(18–21)
Endometrial Cancer	Downregulated	Tumor suppressor; chemosensitizer	Activates FBXO1 transcription	FBXO1, PR, Wnt/β-catenin	Linked to cisplatin sensitivity and tumor differentiation	(22–25)
			Inhibits Wnt/β-catenin			
Gastric Cancer	Downregulated	Metastasis suppressor	Inhibits MMP28	miR-548d-3p, PER1, FNDC5, MMP28	Indirect contributor to anti-metastatic effects	(26–28)
			Part of TPTEP1/miR-548d-3p/PER1 axis			
Hepatocellular Carcinoma	Downregulated	Multifaceted tumor suppressor	Suppresses EMT via Slug feedback	Slug, SAE1, IGFBP4, EMT, oxidative stress markers	Correlates with metastasis; therapeutic potential via EZH2 inhibition	(29–32)
			Maintains redox homeostasis			
Non-Small Cell Lung Cancer	Downregulated	Tumor suppressor; immune modulator	Negatively regulates GADD34	miR-141/660-5p/625-3p/889, GADD34, MDSCs	Potential therapeutic target for miRNA axis modulation	(33–38)
			Inhibits MDSC recruitment			
Glioblastoma	Not mentioned	Tumor suppressor; stemness inhibitor	Synergizes with HDAC inhibitors (LBH589) to induce apoptosis	LBH589, ITGA6, Notch1, OLIG2	Enhances therapy efficacy; reprograms stemness and resistance	(39–42)
			Suppresses Notch1/ITGA6 signaling			
Ovarian Cancer	Downregulated (blood);	Context-dependent roles: Tumor suppressor in metastasis;	Suppresses Notch1/Slug signaling	miR-600, Notch1, Slug, TPD52, miR-223	Blood-based biomarker; potential therapeutic target	(43–46)
	Upregulated (primary tumors)	Pro-survival in primary tumors	Inhibits cancer stemness			
Multiple Myeloma	Upregulated (therapy-responsive)	Tumor suppressor;	Activates NOXA via HDAC inhibition	DANCR, NOXA, Bortezomib, LBH589	Predicts drug response; linked to 9q21 genomic risk locus	(47–49)
		Therapeutic response mediator	Regulated by lncRNA DANCR			
Renal Cell Carcinoma	Downregulated	Tumor suppressor;	KLF9-SNX5 axis suppresses TGF-β/EMT	miR-140-5p, SNX5, CD44, TGF-β	Independent prognostic marker; correlates with immune infiltration	(50–53)
		EMT/metastasis inhibitor	Targeted by miR-140-5p			

## KLF9 and apoptosis of tumor cells

KLF9, as a transcription factor, plays an important role in regulating tumor cell apoptosis. Study have shown that the overexpression of KLF9 significantly enhances the ability of apoptosis, and inhibits the proliferation and migration of tumor cells by regulating the transcription of metallothionein 1m (mt1m) in cholangiocarcinoma cells (64). In addition, KLF9 inhibits the occurrence and development of tumors by activating apoptosis related genes, such as p53. For example, in hepatocellular carcinoma, KLF9 inhibits the proliferation of tumor cells by activating the expression of p53 (65). The relationship between KLF9 and anti apoptotic proteins such as Bcl-2 is also worthy of attention. Bcl-2 is a classic anti apoptotic factor that can prevent the transmission of apoptotic signals in cells. In pancreatic cancer, overexpression of KLF9 can inhibit Bcl-2 and induce cell apoptosis, demonstrating the important role of KLF9 in regulating cell life death balance (54). As a transcription factor, klf9 can also activate tumor cell apoptosis through epigenetic synergistic effects. For example, KLF9 synergistically activates the caspase dependent apoptotic pathway with histone deacetylase inhibitors (such as panobinostat) and induces cell cycle arrest through p21/P27 (39). KLF9 can also inhibit downstream anti apoptotic effects by negatively regulating survival pathways such as PI3K/AKT (15, 66).

## KLF9 and tumor metabolism

KLF9 acts as a transcriptional regulator participating in tumor metabolic control through multiple mechanisms. In endometrial carcinoma cells, suppression of KLF9 significantly upregulates aldehyde-metabolizing enzyme genes ALDH1A1 and AKR7A2, while concurrently inhibiting detoxification genes SULT1A1 and xenobiotic efflux transporter ABCC4, directly disrupting metabolic homeostasis and cell detoxification, which is confirmed in endometrial cancer samples (25). In HCC, SUMO-activating enzyme subunit 1 (SAE1) suppresses KLF9 expression via protein interaction networks, leading to downregulation of the critical acetaldehyde-metabolizing enzyme ALDH2. This induces acetaldehyde metabolic dysfunction and drives tumor metabolic reprogramming, with SAE1-mediated KLF9 inhibition correlating positively with poor prognosis and metastatic risk in HCC patients (31). In breast cancer models, gestational choline nutritional intervention was associated with elevated KLF9 expression in tumor tissues and altered metabolic-related genes (e.g., the oxidative stress regulator TXNIP). This occurs indirectly through epigenetic mechanisms, such as stratifin gene methylation regulating 14-3-3σ protein, thereby inhibiting tumor growth and extending survival (67). Collectively, KLF9 governs metabolic phenotypes either directly by regulating aldehyde-metabolizing enzymes and detoxification pathways, or indirectly by responding to nutritional interventions and upstream regulators via epigenetic modifications. Its dysfunction promotes carcinogenic aldehyde accumulation and impaired detoxification, ultimately driving tumor progression.

## The interaction between KLF9 and the TME

The interaction of KLF9 with the TME is complex and multifaceted, influencing tumor immunity, cancer-associated fibroblasts (CAFs), and angiogenesis. Understanding these interactions is crucial for developing precision therapies and improving patient outcomes in cancer treatment. KLF9 has been shown to modulate immune responses within the TME, interact with CAFs, and affect the formation of new blood vessels, making it a pivotal player in the dynamics of cancer progression and metastasis.

### The role of KLF9 in tumor immune evasion

KLF9 has emerged as a critical regulator of immune evasion mechanisms in tumors. Studies have indicated that KLF9 expression is often downregulated in various cancers, correlating with a more aggressive tumor phenotype and increased metastatic potential (22). This downregulation may enable tumor cells to escape immune surveillance by altering the expression of immune-related genes and modulating the tumor microenvironment. For instance, KLF9 has been implicated in the regulation of immune cell infiltration and the expression of immune checkpoint molecules, which are critical for maintaining immune tolerance in the TME (53). Furthermore, KLF9 interacts with microRNAs to further promote immune evasion by enhancing the proliferation and invasiveness of cancer cells (68). Interestingly, KLF9 plays a significant role in regulating T cell activation, which is crucial for effective immune responses. In an animal study, KLF9 was found to modulate the expression of interferon-related genes and inflammatory cytokines, thereby impacting T cell functionality and their ability to mount an immune response against pathogens or tumors (69). Furthermore, KLF9 is reportedly involved in the maintenance of T cell homeostasis, ensuring a balanced immune response (70).

Current evidence suggests that KLF9 also modulates T cell activation. In immune thrombocytopenia (ITP) patients treated with TPO receptor agonists (TPO-RAs), KLF9 downregulation in MDSCs enhanced immunosuppressive functions by upregulating GADD34 expression. This KLF9-GADD34 axis amplified MDSCs’ capacity to suppress cytotoxic Th1 cells and CD8^+^T cells while promoting the expansion and activity of regulatory T cells (Tregs). Through these mechanisms, KLF9 deficiency in MDSCs reshaped the immune microenvironment, shifting the balance from pro-inflammatory T cell responses toward immune tolerance. Overall, these findings suggest that KLF9 is a pivotal mediator of T cell homeostasis in ITP therapy (71). In nasopharyngeal carcinoma (NPC), KLF9 exhibited a unique pro-tumorigenic role by orchestrating macrophage dysfunction within the tumor microenvironment. KLF9 can transcriptionally activate both HAAO and CYP1B1, driving dual immunosuppressive effects: HAAO induction promotes ferroptosis in M1 macrophages, impairing their antitumor activity, while CYP1B1 overexpression impairs phagocytic capacity in M2 macrophages, facilitating immune escape. This KLF9-mediated reprogramming of macrophage behavior fosters an immunosuppressive niche that accelerates tumor metastasis, contrasting sharply with KLF9’s tumor-suppressive functions observed in other malignancies. The discovery of this mechanism not only reveals KLF9’s context-dependent role in cancer progression but also positions it as a promising therapeutic target, offering potential strategies to reverse macrophage-mediated immunosuppression and combat NPC metastasis through selective pathway modulation (72). KLF9 is also a key player in the regulation of immune checkpoints. Patients with high KLF9 expression (low-risk group) demonstrated enhanced immune microenvironment activity, including elevated infiltration of CD8^+^T cells and NK cells, upregulated antigen-presenting molecules, and increased expression of immune checkpoint markers. This immunologically active phenotype suggests potential sensitivity to immune checkpoint inhibitor therapy and correlates with improved clinical outcomes. Mechanistically, KLF9 may influence tumor immunogenicity by modulating necroptosis-associated signals or indirectly altering chemokine secretion patterns to reshape immune cell recruitment dynamics (59).

### KLF9-dependent regulation of CAF activation

CAFs significantly influence the TME through various mechanisms, including ECM remodeling, secretion of growth factors, and modulation of immune responses. For instance, CAFs secrete matrix metalloproteinases (MMPs) that degrade ECM proteins, facilitating tumor cell invasion and metastasis (73, 74). Besides, CAFs can alter the immune landscape of the TME by secreting immunosuppressive factors that inhibit the activation and proliferation of T cells, thus enabling tumor cells to evade immune surveillance (75, 76). KLF9 also plays a pivotal role in the interaction with CAFs.

The expression of KLF9 in CAFs has been linked to various regulatory mechanisms that modulate the behavior of these fibroblasts in the context of cancer. Studies have shown that KLF9 can act as a transcriptional repressor in CAFs, affecting the expression of genes involved in inflammation, extracellular matrix remodeling, and tumor cell interaction. For instance, KLF9 has been implicated in the downregulation of integrins such as ITGA6 and ITGB1, which are crucial for cell adhesion and migration, suggesting that KLF9 may inhibit the invasive potential of CAFs, thereby limiting their ability to promote tumor metastasis (12). KLF9 exerts its regulatory function via protein-protein interactions, notably through its association with the SIN3A/HDAC epigenetic repressor complex in macrophages. Glucocorticoid-induced KLF9 recruits this complex to suppress immunometabolic gene transcription. This KLF9-SIN3A axis mediates histone deacetylation at promoter regions, silencing M1/M2a polarization markers and inducing macrophage deactivation (8). KLF9 deficiency in CAFs is often associated with increased tumor aggressiveness and poor patient prognosis. For instance, in non-small cell lung cancer, decreased levels of KLF9 correlated with enhanced cell proliferation and invasion, suggesting that KLF9 functions as a tumor suppressor in this context (77). Besides, the regulation of KLF9 is not only limited to microRNAs but also involves epigenetic modifications and interactions with transcriptional co-regulators. The binding of KLF9 to specific DNA sequences in the promoters of target genes can either activate or repress their transcription, depending on the presence of co-factors and the cellular context (78). Accordingly, understanding the regulatory landscape of KLF9 in CAFs is crucial for developing strategies aimed at restoring its expression or function, potentially reversing the pro-tumorigenic effects of CAFs in the tumor microenvironment.

### KLF9 in angiogenesis

Tumors require an adequate blood supply to grow and metastasize, and the angiogenic process is often hijacked by tumor cells to facilitate their own growth. This aberrant angiogenesis is characterized by the release of pro-angiogenic factors, such as vascular endothelial growth factor (VEGF), which promote endothelial cell proliferation and migration (79). The dysregulation of angiogenic signaling pathways often leads to the formation of immature and leaky blood vessels, contributing to a hostile tumor microenvironment that supports cancer progression (80). KLF9 has emerged as a multifunctional regulator of endothelial cell (EC) biology, influencing pathological angiogenesis, metabolic dysregulation, and tumor-associated vascular remodeling. In diabetic retinopathy, hyperglycemia–induced KLF9 overexpression in human retinal microvascular ECs (HRMECs) could transcriptionally activate YAP1 to promote aberrant angiogenesis. KLF9 knockdown reversed this phenotype, suppressing EC proliferation, migration, tube formation, and retinal expression of Ki67/CD31/VEGFA *in vivo*(81). Single-cell analyses of saphenous veins further revealed KLF9’s involvement in metabolic dysfunction-driven proinflammatory signaling within endothelial cells, mediated through metallothionein and IL6^+^EC subsets that disrupted endothelial microenvironments (82). These findings collectively establish KLF9 as a master regulator of endothelial dysfunction through distinct mechanisms: direct YAP1 activation in hyperglycemic conditions, inflammatory pathway modulation in metabolic contexts, and stress-response signaling in tumor vasculature. Therapeutically targeting the KLF9-YAP1 axis may simultaneously inhibit pathological angiogenesis and vascular remodeling. Combining KLF9 modulators with anti-angiogenic agents (e.g., VEGFR2 inhibitors) could address therapeutic resistance in metabolism-driven malignancies. Future research should prioritize mapping KLF9’s spatiotemporal dynamics within tumor microenvironments and elucidating its interplay with hypoxic stress, insights critical for developing precision vascular normalization strategies to impede cancer progression.

### Interaction between KLF9 and signaling pathway

KLF9 plays a significant role in various cellular processes, including development, differentiation, and apoptosis. Its interaction with multiple signaling pathways underscores its importance in regulating cellular functions and its potential implications in cancer biology. Understanding how KLF9 interacts with key signaling pathways such as Wnt, PI3K/Akt, and NF-κB can provide insights into its role in tumorigenesis and therapeutic strategies.

### KLF9 and Wnt Signaling Pathway

**Figure 1:**
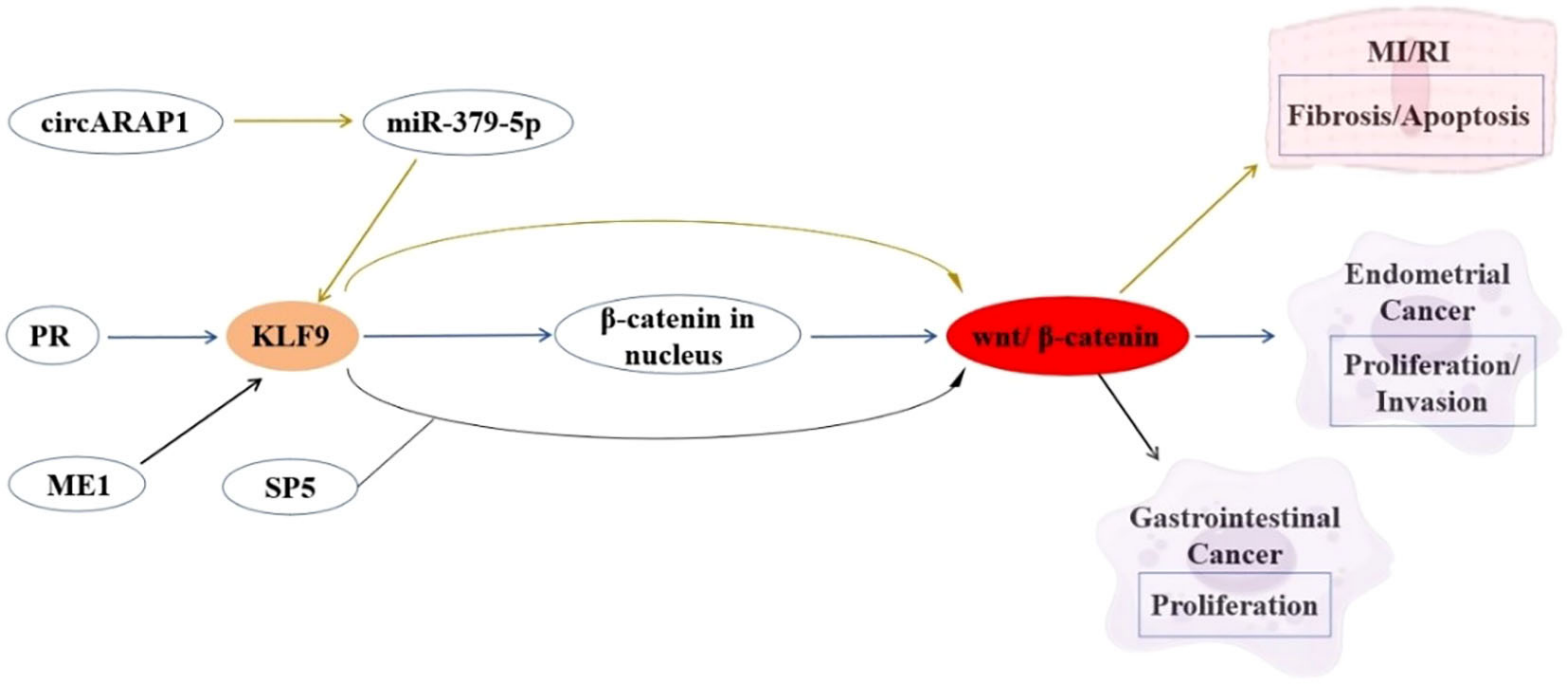
Interaction between KLF9 and Wnt Signaling Pathway Regulatory Axis in Cardiovascular Pathophysiology and Cancer Pathogenesis.

The Wnt/β-catenin signaling pathway plays pivotal roles in cellular homeostasis, cell proliferation, differentiation, and migration. KLF9 functions as a context-dependent modulator of this pathway across various diseases. In myocardial ischemia-reperfusion injury (MI/RI), KLF9 serves as a downstream effector of the circARPA1/miR-379-5p axis, where circARPA1 sequesters miR-379-5p to elevate KLF9 expression. This KLF9 upregulation hyperactivates Wnt/β-catenin signaling, exacerbating cardiomyocyte fibrosis and apoptosis in both MI/RI murine models and hypoxia/reoxygenation-treated cells (83). Conversely, in EC, KLF9 acts as a tumor suppressor by directly inhibiting oncogenic Wnt/β-catenin signaling. KLF9 downregulation in EC correlates with heightened metastatic potential, while its restoration suppresses malignant phenotypes through Wnt pathway repression (22). In gastrointestinal cancer, ME1 overexpression in male ApcMin/+ mice accelerates intestinal tumorigenesis through KLF9-Wnt synergy, while ME1 inhibition suppresses CRC growth (84). KLF9 exhibits tissue-specific Wnt modulation: via miRNA networks in cardiovascular systems versus ME1-driven metabolic reprogramming in CRC (83, 84). Therapeutic approaches include miRNA-based KLF9 silencing for heart protection and ME1 inhibitors with KLF9 restoration for CRC. Future research should clarify KLF9’s spatiotemporal Wnt control and develop tissue-specific modulators that balance physiological Wnt activity with anti-tumor effects, avoiding pathological signaling. These regulatory relationships are summarized in **Figure 1**.

### KLF9 and the PI3K/Akt signaling pathway

**Figure 2:**
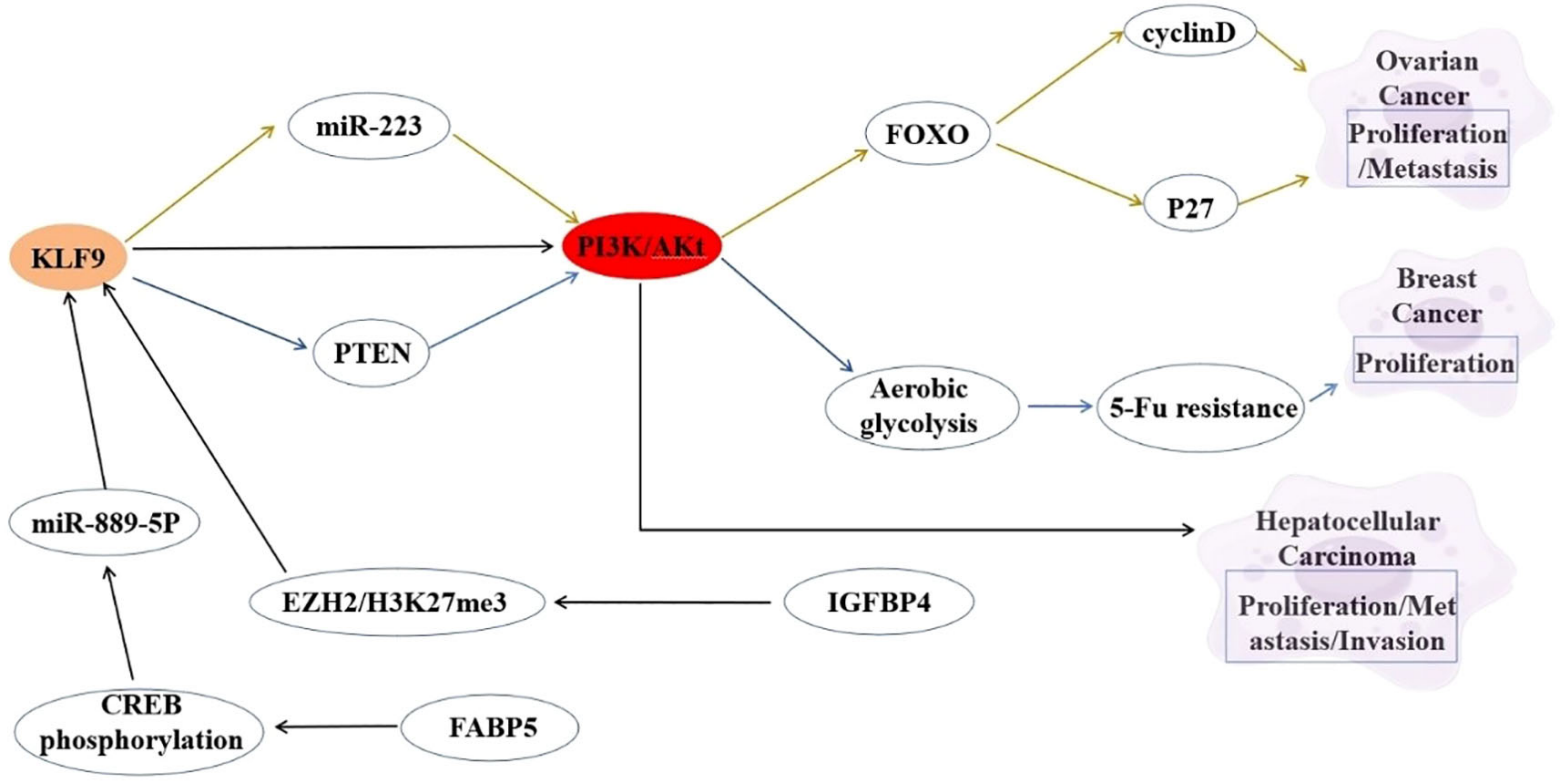
Interaction between KLF9 and PI3K/Akt Signaling Pathway Regulatory Axis in Cancer Pathogenesis.

The PI3K/Akt signaling pathway serves as a master regulator of oncogenic processes, governing cell survival, proliferation, and metabolic reprogramming. KLF9 demonstrates context-dependent functional interplay with this pathway across malignancies. In HCC, KLF9 acts as a tumor suppressor whose downregulation activates PI3K/Akt signaling, driving aggressive proliferation and invasion (85). Bioinformatic analyses in cervical cancer position KLF9 within PI3K/Akt regulatory networks, revealing coordinated gene regulation patterns, despite undefined direct mechanisms (86). The inverse correlation between KLF9 downregulation and PI3K/Akt hyperactivation across malignancies suggests KLF9 may serve as a nodal integrator of oncogenic signaling, potentially through interactions with auxiliary regulators like TPD52/miR-223 and PKCϵ (44). Combining KLF9 expression restoration with PI3K/Akt pathway inhibition may yield synergistic therapeutic effects to counteract pathway dysregulation, while its biomarker potential warrants validation in PI3K/Akt-driven tumor subtypes. Elucidating the mechanistic interplay between KLF9 and PI3K/Akt signaling elements (e.g., PTEN/Akt phosphorylation cascades) is essential to clarify its role as a pathway gatekeeper or collateral target (16, 32). The interaction between KLF9 and the PI3K/Akt signaling pathway is summarized in **Figure 2**.

### KLF9 and the NF-κB signaling pathway

**Figure 3:**
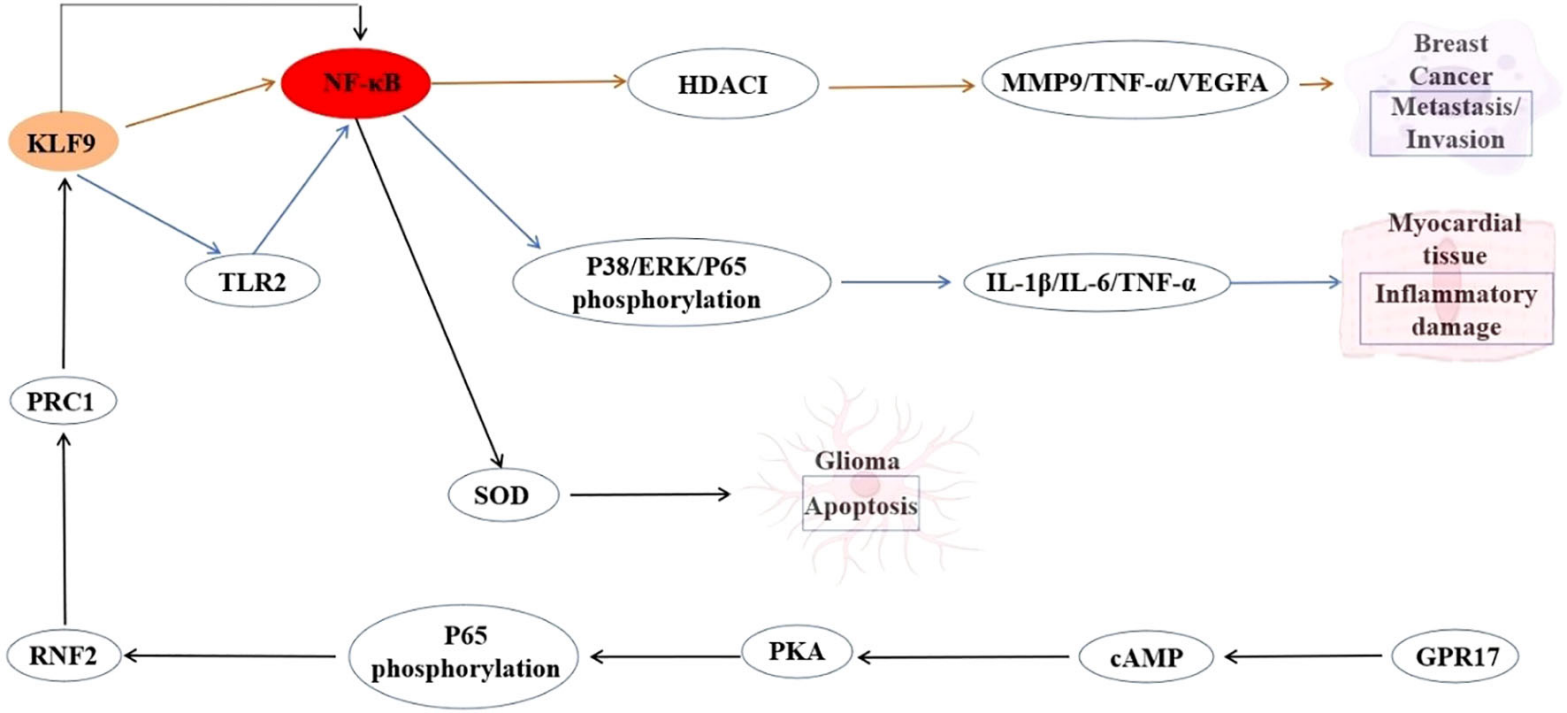
Interaction between KLF9 and NF-κB Signaling Pathway Regulatory Axis in Myocardial Inflammation and Cancer Pathogenesis.

The NF-κB signaling pathway is a critical regulator of inflammation, immune response, and cell survival. KLF9 has been identified as a pro-inflammatory transcription factor in macrophages during myocardial infarction. KLF9 can reportedly drive NF-κB activation by transcriptionally upregulating TLR2 expression through direct binding to its promoter. This KLF9-TLR2 axis can potentiate NF-κB signaling, leading to an increased release of proinflammatory cytokines in macrophages and exacerbating cardiac inflammation. Conversely, suppression of KLF9 can disrupt this cascade, reducing NF-κB activity, mitigating inflammatory damage, and improving cardiac function (87). Another study reported that within the NF-κB-RNF2-KLF9 cascade, KLF9 serves as a key downstream effector mediating GPR17’s antiproliferative and pro-apoptotic roles in glioma pathogenesis, offering a dual targeting strategy involving GPR17 activation or KLF9 modulation (88). KLF9 can also suppress metastasis by transcriptionally repressing NF-κB-driven targets, including MMP9. Beyond MMP9, KLF9 broadly downregulates NF-κB targets, such as TNF-α, VEGFA, and uPA, highlighting its role as a master suppressor of NF-κB-mediated pro-metastatic signaling. This KLF9-NF-κB antagonism underscores a novel tumor-suppressive axis in breast cancer, where KLF9 deficiency promotes NF-κB-driven metastatic programs (5). The interplay between KLF9 and NF-κB exemplifies a dynamic, context-dependent regulatory axis with profound implications across diverse pathological conditions. In inflammatory settings such as myocardial infarction, KLF9 acts as a pro-inflammatory amplifier by directly activating NF-κB via TLR2, driving cytokine storms and tissue damage. Conversely, in cancer, KLF9 has emerged as a tumor-suppressor by inhibiting NF-κB, repressing metastasis-associated genes like MMP9 through chromatin remodeling and direct interference with NF-κB transcriptional activity. These paradoxical roles underscore the therapeutic potential of targeting the KLF9-NF-κB axis, whether by suppressing KLF9 to attenuate inflammation or enhancing its activity to counteract oncogenic NF-κB programs. Future studies should elucidate tissue-specific regulatory mechanisms, and spatiotemporal control of this interplay will be critical for designing precision therapies that harness KLF9’s versatility in balancing NF-κB-driven inflammation, immunity, and malignancy. The interaction between KLF9 and the NF-κB signaling is summarized in **Figure 3**.

## Epigenetic modifications of KLF9 in cancer

Epigenetic modifications play a crucial role in regulating gene expression without altering the underlying DNA sequence. These modifications include DNA methylation, histone modifications, and the involvement of non-coding RNAs, which work synergistically to orchestrate complex gene regulatory networks. The interplay between these epigenetic mechanisms can lead to significant changes in cellular function and has implications for diseases such as cancer, cardiovascular disorders, and neurological conditions. Understanding these modifications, particularly in the context of specific transcription factors like KLF9, can provide novel insights into various biological processes, including development, differentiation, and diseases.

### DNA methylation

KLF9 was identified as one of 13 transcription factors (TFs) exhibiting significant correlations with localized DNA demethylation at their binding sites across 19 cancer types (89). These TFs, enriched for pioneer functions, may regulate chromatin accessibility and modulate methylation patterns. KLF9-bound genomic regions, along with those of SP1, CTCF, NRF1, GABPA, and YY1, were found to exhibit resistance to *de novo*methylation in cancers, likely due to their role in maintaining open chromatin or recruiting epigenetic modifiers. Such methylation dynamics at KLF9-associated sites were linked to immune- and cancer-related pathways. Interestingly, KLF9 may employ analogous mechanisms to enforce localized hypomethylation, positioning it as a key epigenetic regulator in cancer pathology (89). In autoimmune thyroid disease, differential methylation of KLF9 has been observed, suggesting that the methylation landscape can influence the expression of this transcription factor and contribute to disease susceptibility (90). Furthermore, KLF9 has been implicated in the regulation of genes involved in cellular responses to environmental stimuli, and its expression is often correlated with changes in DNA methylation patterns in various cancers, including papillary thyroid carcinoma (58).

### Histone Modifications

KLF9 exhibits context-dependent epigenetic regulation through histone methylation in distinct pathological settings. In cardiac hypertrophy, KLF9 can activate lncRNA UCA1, which recruits the histone methyltransferase EZH2 to catalyze H3K27 trimethylation (H3K27me3) at the p27 promoter, thus epigenetically repressing p27 expression and driving cardiomyocyte hypertrophy (91). Conversely, another study revealed that in olanzapine-induced metabolic dysfunction, KLF9 itself was transcriptionally upregulated via increased H3K4me2 at its gene locus and reduced H3K9me3 mediated by elevated histone demethylases, promoting adipogenic/lipogenic pathways (92). These dual mechanisms highlight KLF9’s dual role as both a regulator and a target of histone methylation, underscoring its centrality in disease-specific epigenetic networks and therapeutic potential.

### Non-coding RNAs regulation

Non-coding RNAs (ncRNAs), particularly lncRNAs and miRNAs, have emerged as significant regulators of gene expression. LncRNAs can function as molecular sponges for miRNAs, thereby modulating the availability of these small RNAs to target mRNAs. For example, lncRNA DANCR has been shown to regulate KLF9 expression by sponging miR-135b-5p, which in turn affects the viability and invasiveness of multiple myeloma cells (47). Similarly, other lncRNAs have been identified to interact with KLF9 and influence its transcriptional activity in various cancer contexts, highlighting the intricacy of regulatory networks involving KLF9 and non-coding RNAs (27, 58). Moreover, miRNAs can directly target KLF9 mRNA, leading to its degradation or translational repression, which adds another layer of complexity to the regulation of this transcription factor (93). The dynamic interplay between ncRNAs and KLF9 exemplifies a sophisticated regulatory axis that fine-tunes oncogenic and tumor-suppressive programs across cancers. These ncRNA-mediated mechanisms not only expand our understanding of KLF9’s contextual regulation but also unveil therapeutic vulnerabilities. Accordingly, modulation of selective ncRNA-KLF9 interactions could enable targeted restoration of KLF9-driven tumor suppression while circumventing inherent genetic or epigenetic constraints.

## KLF9 expression and tumorigenesis

The expression level of KLF9 is closely related to the occurrence of cancer. Specifically, in breast cancer patients, the expression level of KLF9 in cancer tissues was significantly lower than that in normal tissues, and its expression level was related to tumor size and clinical stage (17). Among the tumor tissues of 50 CRC patients, 86% of the samples exhibited lower KLF9 expression compared to normal tissues. This downregulation may be associated with the tumorigenesis process, and the absence of KLF9 protein was confirmed through Western blot analysis and tissue microarrays (18). In pancreatic cancer, KLF9 similarly demonstrates low expression, and this is associated with the degree of differentiation as well as the depth of vascular invasion (54). In ovarian cancer, the expression of KLF9 was abnormally up-regulated. Clinical samples showed that the mRNA and protein levels of KLF9 in tumor tissues were higher than those in normal tissues, while KLF9 knockdown significantly inhibited cell proliferation and the growth of transplanted tumors in nude mice (46). This indicates that although KLF9 is a tumor suppressor in most cancers, its high expression in ovarian cancer may be associated with tumorigenesis, but the mechanism still needs more research. In conclusion, the abnormal expression of KLF9 in different tumor types directly affects the occurrence, metastasis and survival of patients. These clinical studies emphasize the value of KLF9 as a potential biomarker and therapeutic target.

## Discussion

The multifaceted roles of KLF9 in cancer, as outlined in this review, underscore its potential as a pivotal therapeutic target and prognostic biomarker. However, the complexity of its regulatory networks, coupled with context-dependent functional duality, necessitates a comprehensive understanding of its mechanisms and translational implications. Various studies have reported conflicting findings regarding KLF9’s impact on cancer progression, highlighting its complex nature. For instance, in ovarian cancer, KLF9 exhibits heterogeneous expression in tissues (44–46). This duality underscores the need to explore the mechanisms that govern KLF9’s varying roles across different tissues, suggesting that its function may be modulated by other molecular interactions or environmental factors. Current research indicates that KLF9 exerts tumor-suppressive functions through mechanisms operating at multiple levels. At the transcriptional level, it targets and suppresses pro-oncogenic genes while activating tumor-suppressive pathways. Epigenetically, KLF9 maintains a hypomethylated state at key genomic regions and recruits HDAC complexes to silence pro-metastatic genes. Within signaling networks, it cross-regulates core pathways such as Wnt/β-catenin, PI3K-Akt and NF-κB. Regarding the tumor microenvironment, KLF9 remodels the immune landscape and suppresses CAF activation.

However, fundamental limitations exist in current studies: The mechanistic dissection is fragmented, often isolating single pathways for analysis, thereby neglecting KLF9’s core role as a transcriptional-epigenetic-metabolic nexus. For instance, in colorectal cancer, KLF9 concurrently regulates Wnt inhibition and PAD4-mediated metabolic reprogramming, yet the synergistic effects between these actions remain undefined. Furthermore, there is a significant lack of exploration into how SAE1-mediated SUMOylation dynamically regulates KLF9’s transcriptional activity.

The insufficient analysis of dynamic regulation is highlighted by the gap in understanding spatiotemporally specific functional switching mechanisms. In ovarian cancer, the shift of KLF9 from pro-survival in primary tumors to tumor-suppressive in metastases involves microenvironmental stresses (hypoxia/3D mechanical stress) inducing conformational changes and reorganization of cofactor recruitment. However, *in situ*validation techniques for this are lacking. In TNBC, the GR signaling context-driven functional reversal of KLF9 is fundamentally due to differential chromatin accessibility leading to altered recruitment of co-activator complexes. This dynamic process has yet to be tracked at the live-cell level. And single-cell sequencing technology represents a powerful solution to address this challenge since it has revolutionized our understanding of cellular heterogeneity and biological processes at an unprecedented resolution. This technique allows researchers to analyze the genomic, transcriptomic, and epigenomic information of individual cells, which is crucial for deciphering complex biological systems, including cancer. The applications of single-cell sequencing extend across various fields, providing insights into somatic mutations, cell differentiation, and the immune microenvironment. Single-cell sequencing technology enables precise characterization of differential KLF9 expression patterns and functional interactions between malignant and non-malignant cell populations. Furthermore, integrating cryo-electron microscopy to analyze conformational switching mechanisms under microenvironmental stress with computational biology approaches to model the interactive networks across multiple pathways will ultimately elucidate KLF9’s overarching regulatory position in tumor progression.

In addition, the development of agonists/antagonists based on KLF9 is a potential future research direction. Small-molecule agonists and antagonists can provide a more accessible and potentially less invasive means of manipulating KLF9 activity in various diseases. For instance, compounds that augment KLF9 activity could be therapeutically advantageous in conditions characterized by its downregulation, such as certain cancers where KLF9 functions as a tumor suppressor. Conversely, KLF9 antagonists may prove beneficial in pathological contexts where its overexpression drives disease progression. The identification of small molecules that can selectively modulate KLF9 activity is an area of active research. Recent research has shown that the small molecule ONC201 can induce apoptosis in medullary thyroid carcinoma cells by regulating KLF9 among other targets (79). This highlights the potential of small molecule therapies to exploit KLF9’s regulatory roles in cancer and other diseases, paving the way for new treatment paradigms that could be rapidly translated into clinical settings. The combination of intervention measures for KLF9 and existing treatment methods is also a direction of future research. In this respect, the combination of KLF9 inhibitors and conventional chemotherapy drugs may improve the sensitivity of cancer cells to treatment, especially in malignancies where KLF9 promotes tumor survival and proliferation. This synergistic approach could improve clinical outcomes in complex disease states.

KLF9 holds huge potential as a marker for early cancer diagnosis, especially considering its role in regulating cell proliferation, apoptosis, and metastasis. Supporting this, multiple studies have reported substantially reduced KLF9 expression in aggressive cancer cell lines, particularly in prostate and endometrial carcinomas, when compared to non-malignant cells (22, 57). This differential expression suggests that KLF9 could serve as a diagnostic marker, aiding in the identification of early-stage cancers before they progress to more advanced, less treatable stages. Moreover, the ability of KLF9 to inhibit cell growth and induce apoptosis in cancer cells highlights its potential as a therapeutic target, which could be leveraged for early intervention strategies (57). Future research should focus on validating KLF9’s diagnostic capabilities across various cancer types through large-scale clinical trials. Besides, the integration of KLF9 expression profiling with other biomarkers could enhance the accuracy of early cancer detection, ultimately leading to improved patient outcomes.

Although previous studies have shown that KLF9 has potential biomarker and target value in cancer prognosis and treatment, there are still many challenges in its clinical translation process. Firstly, there is significant heterogeneity in the expression of KLF9 across different types of cancer. This means that KLF9 may have different biological functions and clinical significance in different types of cancer or different stages of the same type, therefore individualized strategies are needed to evaluate the effectiveness of KLF9 as a biomarker for different types of cancer.

Then, the function and mechanism of KLF9 still require further research. Although studies have shown that KLF9 can inhibit the proliferation, migration, and invasion of cancer cells, its specific molecular mechanism has not been fully elucidated. For example, KLF9 inhibits the invasiveness of breast cancer cells by regulating the transcription of E-cadherin (14). However, how to translate this mechanism into clinical applications still requires systematic exploration, including the development of KLF9 agonists or inhibitors as therapeutic strategies. Due to the involvement of multiple signaling networks in KLF9 regulation, it increases complexity and may lead to off target effects. At the same time, this multi-level regulation also increases the difficulty of designing pharmacological strategies. The plasticity and compensation mechanism of downstream signaling pathways may also weaken the targeting effect. This highlights the limitations of targeting KLF9, as a single target may not be sufficient to completely block cancer progression. Therefore, combination therapy may be necessary to enhance efficacy, but this further increases the complexity of clinical development.

Furthermore, the clinical application of KLF9 is also limited by detection methods and techniques. Although there are multiple methods to determine the expression of KLF9, such as real-time quantitative PCR and immunohistochemistry, a unified standardized detection process has not yet been established, which may lead to poor comparability of results between different laboratories. In addition, the expression level of KLF9 may be influenced by multiple factors such as the tumor microenvironment and the physiological status of patients, further complicating its application as a clinical biomarker. In addition, the design of clinical trials also faces challenges. The clinical study of KLF9 needs to be conducted in a large-scale patient cohort to validate its reliability and effectiveness as a prognostic marker. However, the heterogeneity of cancer and individual differences among patients may lead to bias in research results, increasing the difficulty of successful transformation. Therefore, when conducting clinical studies related to KLF9, it is necessary to consider the diversity and representativeness of the samples to ensure the broad applicability of the research results.

## Conclusion

The exploration of KLF9 in cancer research has unveiled its significant biological implications and established its crucial role in the complex landscape of tumor biology. As previously mentioned, KLF9 demonstrates context-dependent functionality in cancer. This differential activity suggests regulation by various factors, including cellular background, environmental clues and genetic background, which can significantly affect the function of KLF9. Therefore, future research should prioritize elucidating the specific mechanisms that govern KLF9’s behavior in distinct cancer types and stages. Moreover, the clinical implications of KLF9 are important. The clinical relevance of KLF9 as a biomarker for patient stratification and treatment responsiveness is particularly promising. However, successful translation of these discoveries into clinical practice requires comprehensive assessment of KLF9-targeted intervention strategies through methodically designed preclinical investigations and subsequent validation in controlled clinical trials spanning diverse oncological indications.
